# Underwater Acoustic Signal Acquisition and Sensing Using a Ring Vector Sensor Communication Receiver: Theory and Experiments

**DOI:** 10.3390/s23156917

**Published:** 2023-08-03

**Authors:** Rami Rashid, Erjian Zhang, Ali Abdi

**Affiliations:** Department of Electrical and Computer Engineering, New Jersey Institute of Technology, Newark, NJ 07102, USA; raa62@njit.edu (R.R.); ez7@njit.edu (E.Z.)

**Keywords:** signal acquisition, underwater sensing, underwater sensors, vector sensors

## Abstract

Signal acquisition and sensing in underwater systems and applications is typically a challenging issue due to the small signal strength within the background noise. Here, we present a ring vector sensor communication receiver that can significantly improve signal acquisition, by utilizing the underwater acoustic vector field components, compared to the scalar component. The vector sensor receiver is a multichannel receiver that measures particle velocities, which are vector components of the underwater acoustic field, in addition to the scalar field component. According to the combination of our measured experimental data with our signal acquisition performance analysis, the introduced ring vector sensor receiver exhibits higher signal acquisition probabilities for the vector components compared to the scalar component. This can be attributed to certain characteristics of the vector field components. Another advantage of this multichannel receiver is that combining all of its channels can further increase the signal acquisition and packet detection probability in underwater communication systems compared to a single-channel approach.

## 1. Introduction

Accurate signal acquisition is a fundamental step for the success of signal demodulation in a variety of underwater systems. A useful method for signal acquisition is to append a linearly frequency modulated (LFM) waveform to the beginning of a packet, and then monitor the output of a receiving filter matched to the LFM signal. A sharp peak at the matched filter output marks the beginning of the received packet and allows for successful data demodulation. However, in applications where either noise is strong or signal is weak, the matched filter output does not exhibit a large peak, which can cause high demodulation error.

A vector sensor is a multichannel sensor that simultaneously measures the vector and scalar field components. Given their multichannel nature, vector sensors are used in a variety of applications. Examples include sonar, source localization, angle of arrival estimation, beamforming, and communication [1,2,3,4,5,6,7,8,9,10,11,12,13,14,15,16,17]. The advantages of using a vector sensor for the estimation of the direction of arrival are investigated in [1,2,3]. Analytical results of [4], obtained based on maximizing the directivity index, indicate that the acoustic vector component channels of vector sensors should be utilized for optimal detection. Furthermore, ref. [5] shows that a single vector sensor directivity gain can be four times higher than the directivity of a pressure sensor. Additionally, vector–scalar receivers can be used together with an interferometric method for source detection [6,7,8] and estimating the velocity of a noise source [9]. In addition, the processing gain of vector sensors in noise is addressed in [10], while signal detection using a vertical linear array is investigated in [11].

The papers that utilize a vector sensor as an underwater communication receiver, e.g., [12,13,14,16,17], focus on subject matters such as demodulation, bit error rate, capacity, channel estimation, equalization, etc., rather than signal acquisition performance. More specifically, underwater multichannel equalization using the multiple channels of a vector sensor is proposed in [12]; space–time underwater communication channel parameters are estimated using a vector sensor in [13,14], using a quadrilinear decomposition method, and using MUSIC and ESPRIT algorithms for a multicarrier code-division multiple access systems, respectively; underwater channel capacity bounds are calculated in [16] for a vector sensor receiver; and multi-hop cooperative underwater communication and its bit error rate and capacity are studied in [17] for a frequency-selective channel using an angle-of-arrival model for a vector sensor. However, none of these studies examine the performance of a communication receiver vector sensor at the signal acquisition phase.

The objectives of this paper are: (a) present a ring vector sensor multichannel receiver and study its signal acquisition performance both experimentally and analytically, and (b) compare the multichannel performance with the performance offered by each individual channel of the ring vector sensor. Our solutions to achieve these objectives consist of collecting and analyzing experimental data, combined with mathematical analysis. As demonstrated later, our results allow the examination of the practical feasibility and usefulness of the proposed ring vector sensor receiver for underwater signal acquisition and packet detection.

The rest of this paper is organized as follows. Section 2 provides the definitions for the vector and scalar signals that the ring vector sensor measures. System formulations and performance analysis for multichannel signal acquisition are presented in Section 3. These provide tools and formulas for analyzing the signal acquisition performance of the ring vector sensor receiver using experimental data in Section 4. Concluding remarks are given in Section 5.

## 2. The Ring Vector Sensor Receiver and Its Signals

**Figure 1:**
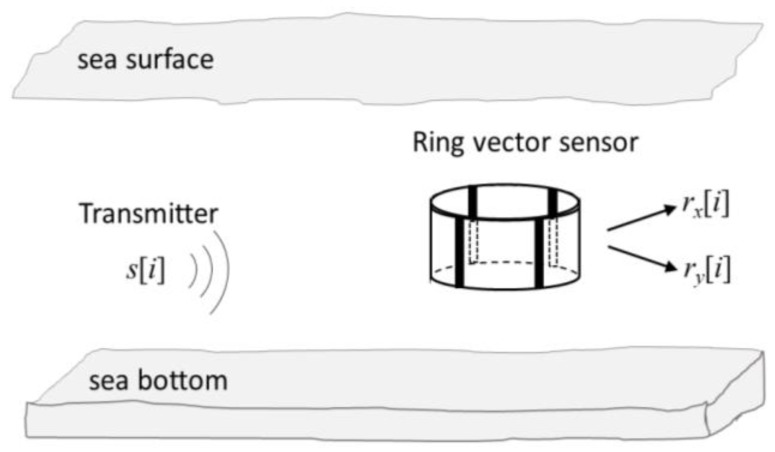
Schematic representation of the ring vector sensor receiver and the two particle velocity signals that it measures in the *x*–*y* plane.

A vector sensor has been found to be useful for multichannel equalization in a communication receiver [12]. Among various designs [18], we consider a ring with four segments, as shown in Figure 1, due to its relative ease of implementation. In response to the transmitted signal s[i], with i being the time index, it measures the two signals given in Equation (1) that are the acoustic particle velocities, i.e., the vector components of the field in the *x*–*y* horizontal plane

(1) $${\left[r_{x}[i]\;r_{y}[i]\;r_{p}[i]\right]}^{T}={\left[h_{x}[i]\;h_{y}[i]\;h_{p}[i]\right]}^{T}⊕s[i]+{\left[n_{x}[i]\;n_{y}[i]\;n_{p}[i]\right]}^{T},$$

where ${}^{T}$ is the transpose. The signal $r_{p}[i]$ in Equation (1) is the acoustic pressure, representing the scalar component of the field, and is measured using a scalar sensor encapsulated in the receiver. The symbol ⊕ in Equation (1) is the convolution; $h_{x}[i]$, $h_{y}[i]$ and $h_{p}[i]$ represent the *x* and *y* acoustic particle velocity and the acoustic pressure channel impulse responses, respectively; and $n_{x}[i]$, $n_{y}[i]$ and $n_{p}[i]$ represent the *x* and *y* acoustic particle velocity and the acoustic pressure noise components, respectively. Note that since the acoustic particle velocity in a particular direction is the spatial gradient of the acoustic pressure in that direction [19], we have the following relations for the *x* and *y* particle velocity components

(2) $$r_{k}[i]=\partial r_{p}[i]/\partial k,\;h_{k}[i]=\partial h_{p}[i]/\partial k,\;n_{k}[i]=\partial n_{p}[i]/\partial k,\;k=x,y.$$

In the next section, Section 3, we consider a multichannel signal acquisition method that can be used to study the signal acquisition performance of the ring vector sensor receiver. A simpler method that is less complex to implement is also considered and studied in Section 3. Comparison and analysis using experimentally measured data are presented in Section 4.

## 3. Multichannel Signal Acquisition

### 3.1. Definitions of Signals, Channels, and Noise

Let s[i],i=0,1,…,N−1, be the signal that is transmitted for acquisition and packet detection at the receiver side. Upon using the three-channel receiver, the received data vector can be written as

(3) r=Sh+n

In this equation, r is a 3N×1 complex vector that represents the received signal plus noise, defined as follows

(4) $$r={\left[r^{T}[0]r^{T}[1]\cdots \;r^{T}[N-1]\right]}^{T},$$

(5) $$r[i]={\left[r_{1}[i]\;r_{2}[i]\;r_{3}[i]\right]}^{T},i=0,1,\ldots ,N-1,$$

where the three channels of the receiver are labeled 1, 2, 3, instead of *x*, *y*, *p*, for notational convenience. In the above equations, S is a 3N×3 matrix that is composed of *N* signal samples, as follows

(6) $$S=s⊗I_{3},\;s={\left[s[0]s[1]\cdots s[N-1]\right]}^{T},$$

where ⊗ is the Kronecker product, $I_{3}$ is the 3×3 identity matrix, and h represents the underwater channel responses sensed by the three-channel receiver

(7) $$h={\left[h_{1}\;h_{2}\;h_{3}\right]}^{T},$$

with the following covariance matrix

(8) $$\Sigma_{h}=E[hh^{†}]=\mathrm{diag}(\eta_{1}^{2},\eta_{2}^{2},\eta_{3}^{2}),$$

where *E* is the mathematical expectation, ${}^{†}$ is the transpose conjugate and diag stands for the diagonal matrix. Additionally, **n** in (3) is a 3N×1 complex vector that represents the additive noise observed by the three-channel receiver

(9) $$n={\left[n^{T}[0]n^{T}[1]\cdots n^{T}[N-1]\right]}^{T},$$

(10) $$n[i]={\left[n_{1}[i]n_{2}[i]n_{3}[i]\right]}^{T},\;i=0,1,..,N-1,$$

with the following covariance matrix

(11) $$\Sigma_{n}=E[n[i]n^{†}[i]]=\mathrm{diag}(\sigma_{1}^{2},\sigma_{2}^{2},\sigma_{3}^{2}).$$

In Section 3.2 below, we consider and analyze a multichannel signal acquisition method to understand the signal acquisition performance of the ring vector sensor receiver, followed by considering and analyzing a simpler method in Section 3.3 that has lower implementation complexity.

### 3.2. Multichannel Combining

**Figure 2:**
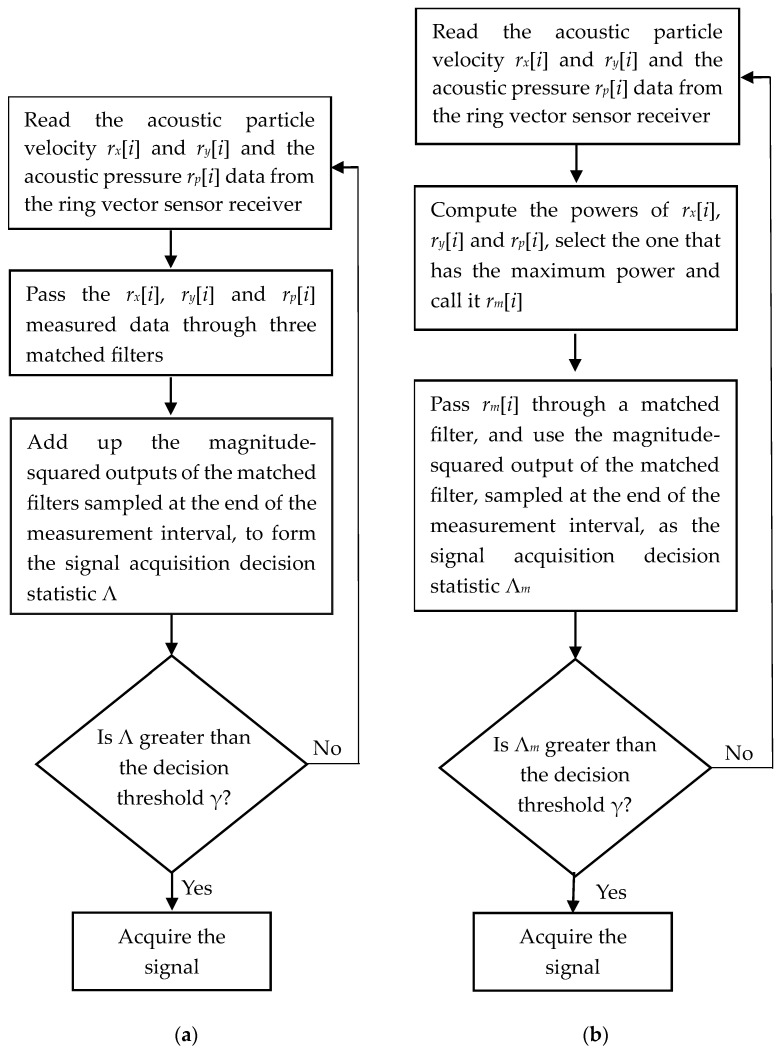
Flowcharts for the presented methods: (**a**) multichannel combining, (**b**) maximum power selection.

Matched filtering is a useful approach for acquisition and packet detection in communication receivers. By appending a known signal s[i], such as an LFM signal, to the beginning of the packet and then monitoring the output of a receiving filter matched to the signal, acquisition can be accomplished. To analyze the signal acquisition performance of the multichannel ring vector sensor receiver, we consider a multichannel combining approach where each channel of the vector sensor is fed into a matched filter, matched to s[i], and then the outputs of the three matched filters are combined. If $z_{k}$ denotes the output of the *k*th matched filter sampled at i=N−1, then the signal acquisition decision statistic Λ of the ring vector sensor receiver can be written as

(12) $$\Lambda =\sum_{k=1}^{3}\Lambda_{k},\;\Lambda_{k}{=}^{}|z_{k}{|}^{2},\;z_{k}=\sum_{i=0}^{N-1}s^{*}[i]r_{k}[i],$$

where ${}^{*}$ represents the conjugate. A flowchart of this method is presented in Figure 2a. Following the Neyman-Pearson theorem [20], the signal is successfully acquired with probability $P_{A}$, if Λ>γ, where γ is the decision threshold determined by the probability of false acquisition $P_{FA}$. Since *N* is usually large, $z_{k}$ in (12) can be considered to follow a complex normal distribution, based on the central limit theorem. Let $H_{1}$ and $H_{0}$ represent the signal-in-noise and noise-only scenarios, respectively. Additionally, $\rho_{k}=E[|z_{k}{|}^{2}|H_{1}]=(d_{k}+1)\sigma_{k}^{2}\bar{E}$ and $\xi_{k}=E[|z_{k}{|}^{2}|H_{0}]=\sigma_{k}^{2}\bar{E}$, where $d_{k}=(\eta_{k}^{2}/\sigma_{k}^{2})\bar{E}$ and $\bar{E}$ is energy of the signal s[i]. The characteristic function (CF) of $\Lambda_{k}$ in (12), $\Theta_{\Lambda_{k}}(\omega )=E[\exp(j\omega \Lambda_{k})]$ with $j^{2}=-1$, can be shown to be ${\left(1-j\rho_{k}\omega \right)}^{-1}$ and ${\left(1-j\xi_{k}\omega \right)}^{-1}$ under $H_{1}$ and $H_{0}$, respectively. Upon applying partial fraction expansion to the CF of Λ, $\Theta_{\Lambda }(\omega )=\prod_{k=1}^{3}\Theta_{\Lambda_{k}}(\omega )$, followed by inverse Fourier transform to obtain probability density function (PDF) from CF [20], PDF of Λ can be obtained and integrated, which result in the following performance probabilities

(13) $$P_{A}=\mathrm{Pr}(\Lambda >\gamma |H_{1})=\sum_{k=1}^{3}U_{k}\exp(-\gamma /\rho_{k}),$$

(14) $$P_{FA}=\mathrm{Pr}(\Lambda >\gamma |H_{0})=\sum_{k=1}^{3}V_{k}\exp(-\gamma /\xi_{k}).$$

In the above equations, we have $U_{k}=\prod_{k^{\prime }=1,k^{\prime }\neq k}^{3}(1-\rho_{k^{\prime }}\rho_{k}^{-1}{)}^{-1}$ and $V_{k}=\prod_{k^{\prime }=1,k^{\prime }\neq k}^{3}(1-\xi_{k^{\prime }}\xi_{k}^{-1}{)}^{-1}$ as the partial fraction expansion coefficients of $\Theta_{\Lambda }(\omega )$ under $H_{1}$ and $H_{0}$, respectively.

To implement this multichannel signal acquisition method for the ring vector sensor receiver, three matched filters are needed. In the next subsection, we consider a simpler method that requires only one matched filter to implement, and then compare its performance with the implementation that needs three matched filters.

### 3.3. Maximum Power Selection

Let $\Omega_{k}=E[|r_{k}[i]{|}^{2}]$, k=1,2,3, be the power of $r_{k}[i]$. In addition, let $r_{m}[i]$ denote the one that has the maximum power, i.e., $\Omega_{m}=E[|r_{m}[i]{|}^{2}]=\max\{\Omega_{1},\;\Omega_{2},\;\Omega_{3}\}$. The signal acquisition decision statistic $\Lambda_{m}$ in response to this selected signal $r_{m}[i]$ is given by

(15) $$\Lambda_{m}{=}^{}|z_{m}{|}^{2},\;z_{m}=\sum_{i=0}^{N-1}s^{*}[i]r_{m}[i],$$

where $z_{m}$ is the output of the matched filter for the strongest receiver channel, sampled at i=N−1. A flowchart of this method is presented in Figure 2b. Following the same approach as the previous subsection, the following performance probabilities can be obtained for this detector

(16) $$P_{A,m}=\mathrm{Pr}(\Lambda_{m}>\gamma |H_{1})=\exp(-\gamma /\rho_{m}),$$

(17) $$P_{FA,m}=\mathrm{Pr}(\Lambda_{m}>\gamma |H_{0})=\exp(-\gamma /\xi_{m}),$$

where $\rho_{m}=E[|z_{m}{|}^{2}|H_{1}]=(d_{m}+1)\sigma_{m}^{2}\bar{E},$ $d_{m}=(\eta_{m}^{2}/\sigma_{m}^{2})\bar{E}$ and $\xi_{m}=E[|z_{m}{|}^{2}|H_{0}]=\sigma_{m}^{2}\bar{E}$.

To theoretically compare the performance of the maximum power selection signal acquisition method with the multichannel combining method, first we consider the case where the noise powers are equal, i.e., $\sigma_{1}^{2}=\sigma_{2}^{2}=\sigma_{3}^{2}=\sigma^{2}$ and consequently $\xi_{1}=\xi_{2}=\xi_{3}=\xi$ (this assumption is dropped in the next section). Then, we obtain this CF for Λ under $H_{0}$, $\Theta_{\Lambda }(\omega )={\left(1-j\xi \omega \right)}^{-3}$, which, upon inverse Fourier transform and integration of the resulting PDF, a chi-squared PDF [20] with six degrees of freedom, provides the following probability

(18) $$P_{FA}=\exp(-\gamma /\xi )(1+(\gamma /\xi )+0.5{\left(\gamma /\xi \right)}^{2}).$$

**Figure 3:**
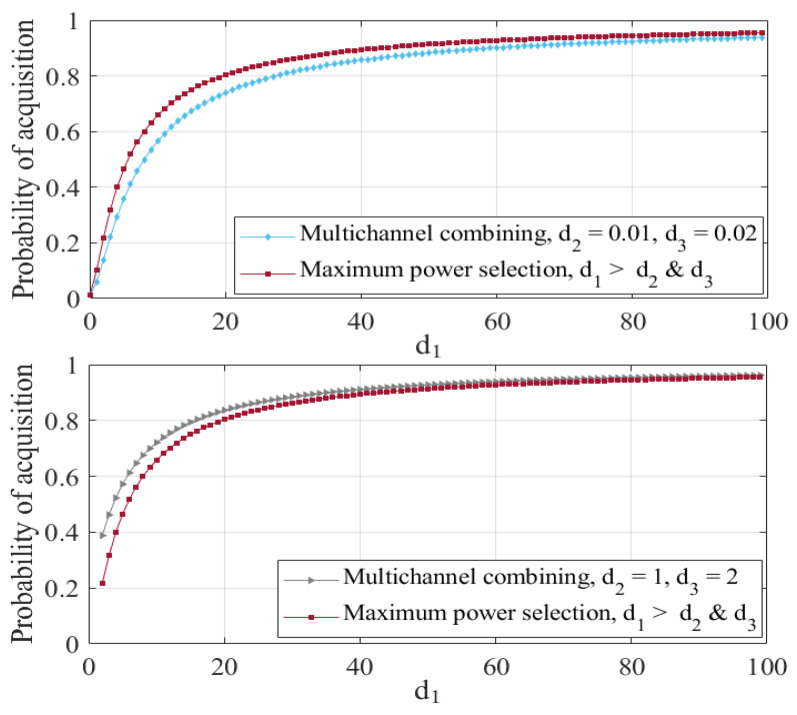
Probability of signal acquisition for the two methods.

For any given value of $P_{FA}$, Equation (18) can be solved and the resulting γ needs to be substituted in (13) to compute $P_{A}$ for the multichannel combining method. For the maximum power selection method and by combining (16) and (17), we obtain, $P_{A,m}=P_{FA,m}^{1/(d_{m}+1)}.$ Using these results, the signal acquisition probabilities of these two methods are graphed and compared in Figure 3, where, without loss of generality, it is assumed that receiver channel 1 has the maximum power.

Figure 3 shows $P_{A}$ and $P_{A,m}$ plotted as functions of $d_{1}$, signal-to-noise ratio (SNR) of the receiver channel 1, with $\left(d_{2},d_{3})=(0.01,0.02\right)$ in the top panel and $\left(d_{2},d_{3})=(1,2\right)$ in the bottom panel, $P_{FA}=0.01$, and unit energy signal. We note that when channels 2 and 3 are not strong (top panel), the maximum power selection method that picks channel 1 outperforms the multichannel combining method, which is a reasonable result. When channels 2 and 3 become stronger (bottom panel), the selection method converges to the combining method, as $d_{1}$ increases.

## 4. Analysis Using Experimental Data

### 4.1. The Setup for Measurements and Experiments

To study the signal acquisition performance of the ring vector sensor receiver employing the selection method or the combining method, underwater experiments were conducted. More specifically, one hundred LFM signals were transmitted in a large pool. Experiments were conducted along the length of the pool, which was about 23 × 13 m in size, and 1–3 m in depth. The transmitter and receiver were submerged in water and were separated by 20 m. They were 0.6 m below the water surface. During the experiments, there were some swimming activities in some other lanes. The LFM signal was generated in complex baseband, that is, $s[i]=\exp[j2\pi B_{0}{\left(2T_{0}\right)}^{-1}{\left(i/f_{s}\right)}^{2}]$, where $B_{0}$ represents the bandwidth, $T_{0}$ represents the signal duration, and $f_{s}$ is the sampling rate. Then it was converted to a real passband waveform before the transmission. The duration and bandwidth of each transmitted LFM signal were $T_{0}=0.2$ s and 8 kHz, centered at 20 kHz. The spacing between each two consecutive LFM signals was also $T_{0}$. An omnidirectional transducer was used as the transmitter. On the receiver side, the ring vector sensor receiver that was used simultaneously measured the x and y components of the acoustic vector field, i.e., the acoustic particle velocity, and the p scalar component of the acoustic field, i.e., the acoustic pressure.

The data measured by all the channels of the ring vector receiver were analog real passband waveforms that were fed into an analog-to-digital converter (ADC) with the sampling rate of $f_{s}=$ 100,000 samples per second. The ADC was connected to a laptop in which the collected data were stored. To prepare the data for the analysis conducted in the next subsection and using the methods and equations presented in Section 3, the data were converted to the complex baseband format.

### 4.2. Data Analysis

**Figure 4:**
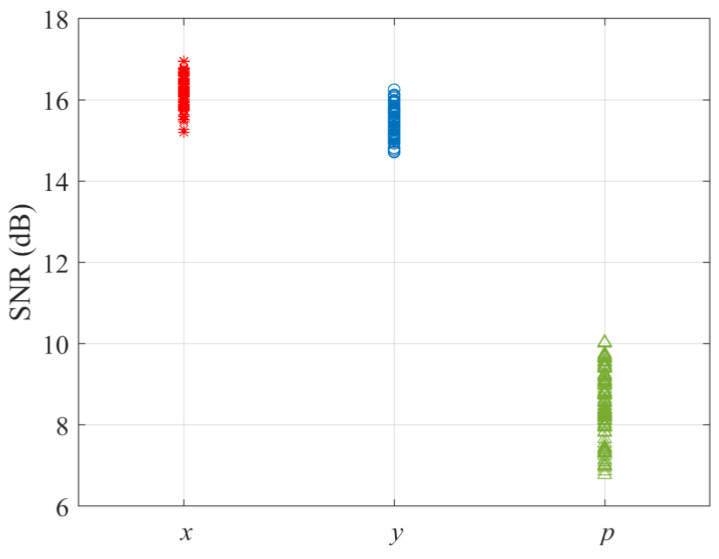
Measured vector and scalar signal-to-noise ratios.

**Figure 5:**
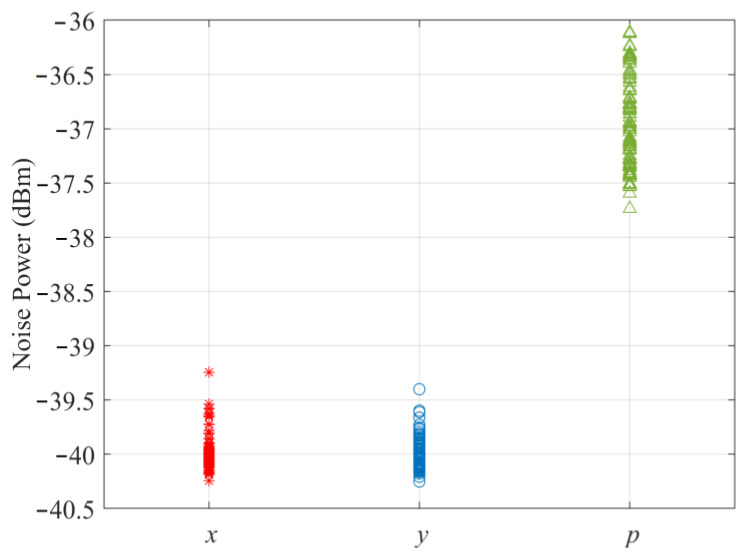
Measured vector and scalar noise powers.

**Figure 6:**
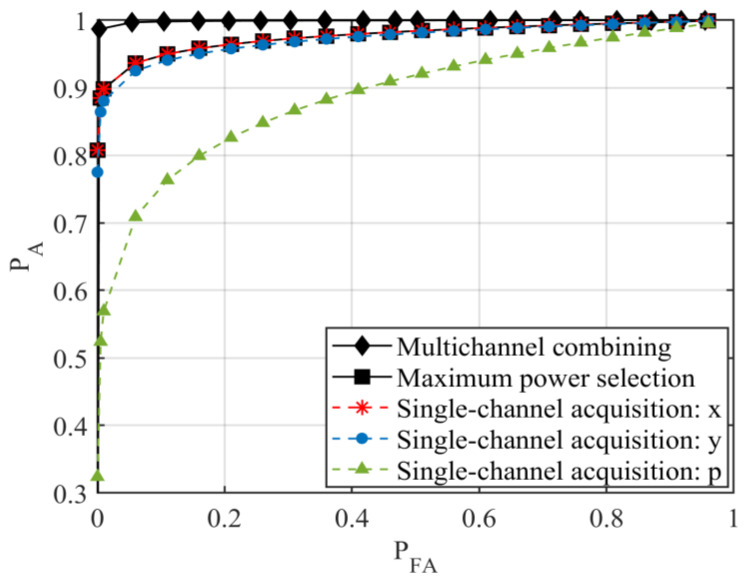
Experimental signal acquisition performance curves for the ring vector sensor receiver.

To evaluate the acquisition performance of the multichannel combining ring vector sensor receiver using experimental data, SNRs and noise powers for various channels are first calculated from one hundred trials and are all shown in Figure 4 and Figure 5, respectively. For the *k*th receiver channel, the averages of the hundred measured SNRs and noise powers are used to determine $d_{k}$ and $\sigma_{k}^{2}$, respectively, which in turn specify numerical values for $\rho_{k}$ and $\xi_{k}$, respectively. By substituting these in (13) and (14), *P_A_* versus *P_FA_* receiver operating characteristic curve for the ring vector sensor receiver using the multichannel combining method is obtained and graphed in Figure 6, together with the *P_A_* versus *P_FA_* curve for each individual channel of the ring vector sensor receiver (results of the maximum power selection method are discussed at the end of this subsection).

We observe that the multichannel combining method for the ring vector sensor receiver offers the highest signal acquisition probability compared to each of its individual channels. Additionally, the *x* and *y* channels appear to exhibit higher signal acquisition probabilities than the *p* channel. To understand why the *x* and *y* channels outperform the *p* channel, we look at the measured SNRs. Figure 4 shows one hundred experimental data points for the SNR of each of the x and y vector components and the p scalar component measurements of the ring vector sensor receiver. We note that the SNRs of the vector components are higher than those of the scalar components. This can be attributed to the smaller noise powers of the vector components, according to the hundred noise measurements shown in Figure 5 for each component. A theoretical explanation for the lower noise powers of the vector components is that they do not receive noise from all directions, whereas the scalar component collects noise from various directions [12].

For the maximum power selection method, the *P_A_* versus *P_FA_* curve is shown in Figure 6, obtained using (16) and (17) and the experimental data. We notice that the selection method and the combining method exhibit a nearly similar performance. This is because there is at least one strong channel that individually provides high *P_A_*, and adding more channels slightly increases *P_A_*.

## 5. Conclusions

In this paper, a new receiver for packet detection and signal acquisition in underwater communication and sensing applications is proposed. The proposed receiver is a ring vector sensor receiver that benefits from the multiple signals that it collects from the field. These signals are particle velocities that are vector components of the field, in addition to the scalar field component. Our experimental measurements, along with mathematical analysis, demonstrate that the signal acquisition probabilities of the vector components are higher compared to the scalar component. According to the experimental data, this could be related to the higher SNRs and lower noise powers of the vector field components. Additionally, the multichannel combining method that uses all of the channels of the ring vector sensor receiver offers the highest signal acquisition probability compared to each of its individual channels. Moreover, it is demonstrated that if the receiver channels are strong, using the strongest channel can provide nearly the same acquisition performance as the multichannel combining method, while being less complex to implement.

To theoretically investigate how the angular behavior of the vector sensor receiver may affect its signal acquisition performance, the interested reader can refer to the analysis presented in Appendix A.

Given the recent advances in distributed underwater acoustic sensor networks and their applications, a future research topic could be investigating signal acquisition in such networks using multichannel vector sensors.

## Figures and Tables

**Figure A1:**
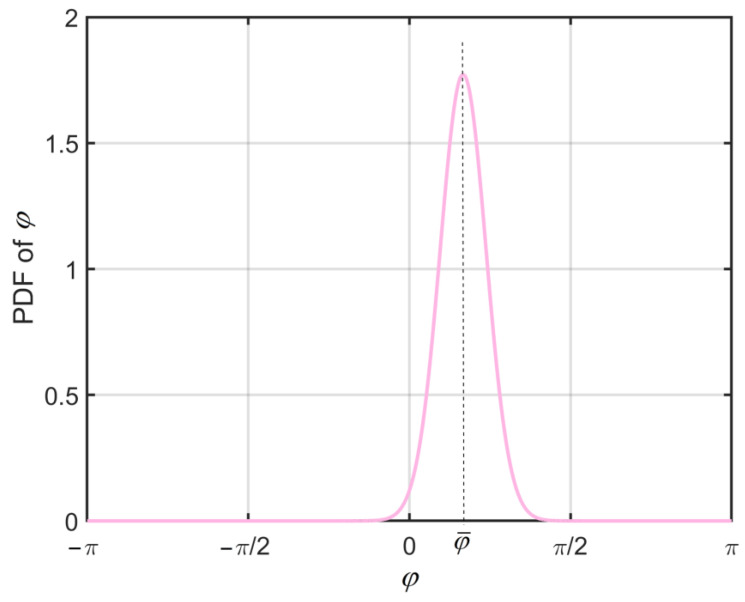
The angular von Mises probability density function.

**Figure A2:**
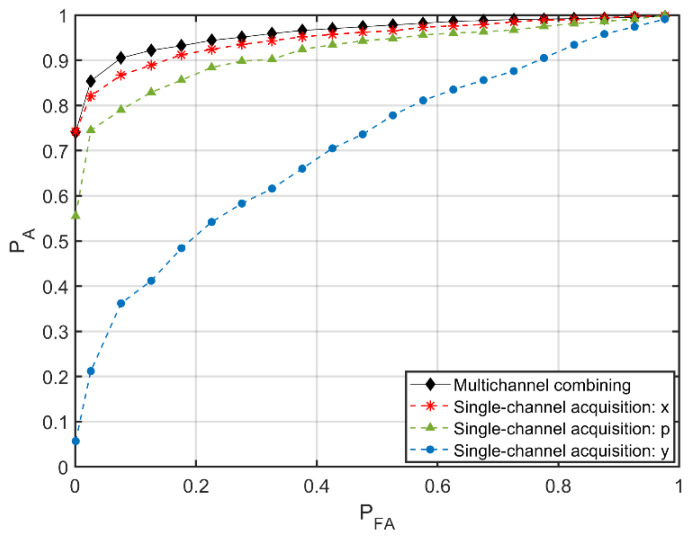
Simulated signal acquisition performance curves when the transmitter is close to the *x* axis.

**Figure A3:**
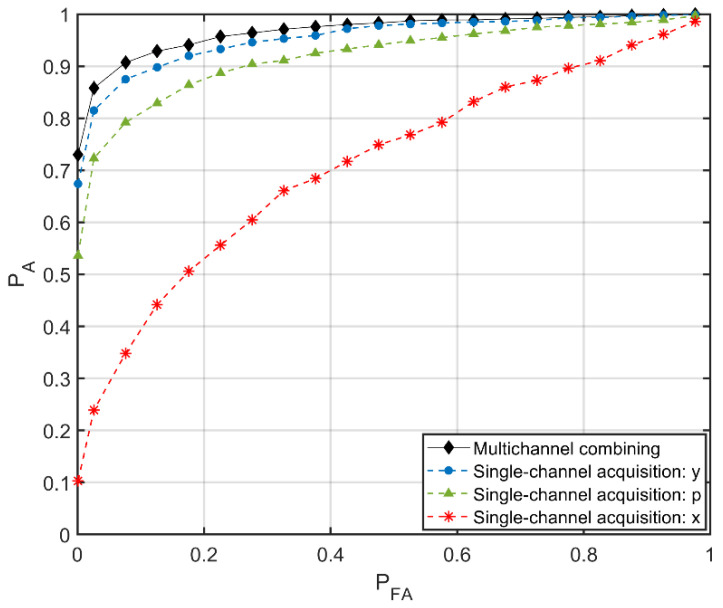
Simulated signal acquisition performance curves when the transmitter is close to the *y* axis.

## Appendix A. Analysis of the Angular Dependence of the Signal Acquisition Performance

Given the angular selectivity of a vector sensor, the transmitter position (transmitter angle) changes the power distribution among the channels of the vector sensor receiver [4]. To understand how the transmitter position affects the signal acquisition performance studied in this paper, suppose the transmitter is located at the angle $\bar{\varphi }$ in the x−y plane. Using the angular von Mises PDF [21] for the signal angle-of-arrival φ around the mean $\bar{\varphi }$

(A1) $$f(\varphi )=\frac{\exp(\nu \cos(\varphi -\bar{\varphi }))}{2\pi I_{0}(\nu )},\;\nu \geq 0,\;-\pi <\bar{\varphi }<\pi ,$$

we can obtain the closed-form expressions for the *x* and *y* channel powers that we need in this study. In the above PDF, ν controls the width of the PDF and $I_{\ell }(.)$ is the ℓ*-*th order modified Bessel function of the first kind. An example of this PDF for $\bar{\varphi }=30^{{}^{\circ}}$ and ν=20 is shown in Figure A1. The von Mises PDF is widely used for the characterization and analysis of angular variables, and includes or approximates several other angular PDFs [22]; moreover, given its mathematical form, it can result in useful closed-form formulas for channel correlation functions and channel powers of the vector sensor, as demonstrated below.

Let $h_{p}(x_{0},y_{0})$ represent the acoustic pressure at a point $\left(x_{0},y_{0}\right)$. By definition, the spatial correlation function of $h_{p}$ is given by $c_{h_{p}}(\Delta x,\Delta y)=E[h_{p}(x_{0},y_{0})h_{p}^{*}(x_{0}+\Delta x,y_{0}+\Delta y)]$. Upon writing $h_{p}(x_{0},y_{0})$ and $h_{p}(x_{0}+\Delta x,y_{0}+\Delta y)$ as superpositions of plane waves received at the points $\left(x_{0},y_{0}\right)$ and $\left(x_{0}+\Delta x,y_{0}+\Delta y\right)$ in the two-dimensional plane, respectively, it can be shown that $c_{h_{p}}(\Delta x,\Delta y)=\eta_{p}^{2}E_{\varphi }[\exp\{j\kappa \sqrt{{\left(\Delta x\right)}^{2}+{\left(\Delta y\right)}^{2}}\cos(\varphi -\varphi_{0})\}]$ [23], where $\eta_{p}^{2}=E[|h_{p}{|}^{2}]$ is the power of $h_{p}$, $\varphi_{0}=\tan^{-1}(\Delta y/\Delta x)$, κ=2π/λ and λ is the wavelength. By substituting f(φ) of (A1) and computing the mathematical expectation with respect to φ using [24], we obtain the following spatial correlation function

(A2) $$c_{h_{p}}(\Delta x,\Delta y)=\eta_{p}^{2}\frac{J_{0}\left(j\sqrt{\nu^{2}-\kappa^{2}({\left(\Delta x\right)}^{2}+{\left(\Delta y\right)}^{2})+j2\nu \kappa (\Delta x\cos\bar{\varphi }+\Delta y\sin\bar{\varphi })}\right)}{J_{0}(j\nu )}$$

where $J_{\ell }(0)$ is the ℓ-th order Bessel function of the first kind and $I_{\ell }(\zeta )=j^{-\ell }J_{\ell }(j\zeta )$. Since the acoustic particle velocity *x* and *y* components are the spatial gradients of the acoustic pressure along the *x* and *y* axes, that is, $h_{x}=\partial h_{p}/\partial x$ and $h_{y}=\partial h_{p}/\partial y$, taking certain derivatives of $c_{h_{p}}(\Delta x,\Delta y)$ in (A2), according to the formulas presented in [23], provides the following results for the vector sensor channel powers and correlations

(A3) $$\begin{array}{lll} \eta_{x}^{2} & = & E[|h_{x}{|}^{2}]=-\kappa^{-2}\partial^{2}c_{h_{p}}(\Delta x,\Delta y=0)/\partial {\left(\Delta x\right)}^{2}|{}_{\Delta x=0},\\ & = & \eta_{p}^{2}\left\{\frac{1}{2}\frac{J_{0}(j\nu )-J_{2}(j\nu )}{J_{0}(j\nu )}\cos^{2}\bar{\varphi }+\frac{j(\cos^{2}\bar{\varphi }-1)}{\nu }\frac{J_{1}(j\nu )}{J_{0}(j\nu )}\right\}, \end{array}$$

(A4) $$\begin{array}{lll} \eta_{y}^{2} & = & E[|h_{y}{|}^{2}]=-\kappa^{-2}\partial^{2}c_{h_{p}}(\Delta x=0,\Delta y)/\partial {\left(\Delta y\right)}^{2}|{}_{\Delta y=0},\\ & = & \eta_{p}^{2}\left\{\frac{1}{2}\frac{J_{0}(j\nu )-J_{2}(j\nu )}{J_{0}(j\nu )}\sin^{2}\bar{\varphi }+\frac{j(\sin^{2}\bar{\varphi }-1)}{\nu }\frac{J_{1}(j\nu )}{J_{0}(j\nu )}\right\}, \end{array}$$

(A5) $$\begin{array}{lll} E[h_{x}h_{y}^{*}] & = & -\kappa^{-2}\partial^{2}c_{h_{p}}(\Delta x,\Delta y)/\partial \Delta x\partial \Delta y|{}_{\Delta x=\Delta y=0},\\ & = & \eta_{p}^{2}\left\{\frac{1}{2}\frac{J_{0}(j\nu )-J_{2}(j\nu )}{J_{0}(j\nu )}\sin\bar{\varphi }\cos\bar{\varphi }+\frac{j\sin\bar{\varphi }\cos\bar{\varphi }}{\nu }\frac{J_{1}(j\nu )}{J_{0}(j\nu )}\right\}, \end{array}$$

(A6) $$\begin{array}{lll} E[h_{p}h_{x}^{*}] & = & {\left(j\kappa \right)}^{-1}\partial c_{h_{p}}(\Delta x,\Delta y=0)/\partial \Delta x|{}_{\Delta x=0},\\ & = & -j\eta_{p}^{2}\frac{J_{1}(j\nu )}{J_{0}(j\nu )}\cos\bar{\varphi }, \end{array}$$

(A7) $$\begin{array}{lll} E[h_{p}h_{y}^{*}] & = & {\left(j\kappa \right)}^{-1}\partial c_{h_{p}}(\Delta x=0,\Delta y)/\partial \Delta y|{}_{\Delta y=0},\\ & = & -j\eta_{p}^{2}\frac{J_{1}(j\nu )}{J_{0}(j\nu )}\sin\bar{\varphi }. \end{array}$$

Using Equations (A3) and (A4) and this property of Bessel functions $\zeta J_{2}(\zeta )=2J_{1}(\zeta )-\zeta J_{0}(\zeta )$ [24], it can be shown that $\eta_{x}^{2}+\eta_{y}^{2}=\eta_{p}^{2}$. Since the sum of the *x* and *y* particle velocity channel powers is constant, it means that as $\bar{\varphi }$, which specifies the transmitter position, increases counterclockwise with respect to the positive *x* axis, the *x*-channel power $\eta_{x}^{2}$ decreases, whereas the *y-*channel power $\eta_{y}^{2}$ increases. To see how this affects the signal acquisition performance of the ring vector sensor receiver, we produce $P_{A}$ versus $P_{FA}$ performance curves via simulations, as explained below.

**Numerical Simulations:** We consider an isotropic noise field [4,12] that renders the noise covariance matrix of $\Sigma_{n}=\mathrm{diag}(\sigma_{x}^{2},\sigma_{y}^{2},\sigma_{p}^{2})$ for the three-channel vector sensor receiver, where $\sigma_{x}^{2}=\sigma_{y}^{2}=\sigma_{p}^{2}/2$. Then, we generate 1000 complex normal realizations of the 3N×1 complex noise vector n in (9) that includes N samples of the noise for each of the three channels of the vector sensor receiver. Then, using (12), we simulate 1000 values for the decision statistic Λ that, for a given $P_{FA}$, allows the computation of the corresponding decision threshold γ such that $P_{FA}=\mathrm{Pr}(\Lambda >\gamma |H_{0})$ and $H_{0}$ represents the noise-only scenario. To compute $P_{A}$ for this γ according to $P_{A}=\mathrm{Pr}(\Lambda >\gamma |H_{1})$, where $H_{1}$ represents the signal-in-noise scenario, we need to simulate 1000 values for the decision statistic Λ under $H_{1}$ and using (12) as well. Therefore, 1000 realizations of the 3N×1 complex vector r=Sh+n of signal plus noise in (3) are also generated, where r includes N samples of the received LFM signal plus noise for each of the three channels of the vector sensor receiver. Here, we note that based on (6), the 3N×3 complex matrix S is composed of *N* LFM signal samples, where the LFM signal and its parameters are given in Section 4.1. Moreover, to generate the realizations of r, we generate 1000 complex normal realizations of the three channels of the vector sensor receiver $h={\left[h_{x}h_{y}\;h_{p}\right]}^{T}$ with the covariance matrix of $\Sigma_{h}=E[hh^{†}]$, for which the elements are given by Equations (A3)–(A7). By repeating the above simulation steps for the $P_{FA}$ values shown in Figure A2 and Figure A3, the associated $P_{A}$ values are computed and used to graph the performance curves presented in these two figures. The considered numerical values for the parameters in the simulations are $\sigma_{p}^{2}=0.1$, N=1000, ν=20, and $\bar{\varphi }=10^{o}$ or $80^{o}$, which indicates that the transmitter is close to the *x* axis or the *y* axis, respectively.

The simulated $P_{A}$ versus $P_{FA}$ curve for each individual channel of the ring vector sensor receiver is shown in Figure A2, when the transmitter is close to the *x* axis. We observe that the acquisition performance of the *x* channel is better than that of the *y* channel. This can be attributed to the higher SNR of the *x* channel, $10\log_{10}(\eta_{x}^{2}/\sigma_{x}^{2})=12.7\;\mathrm{dB}$, compared to the SNR of the *y* channel, $10\log_{10}(\eta_{y}^{2}/\sigma_{y}^{2})=2\;\mathrm{dB}$. The situation is reversed and the *y* channel performance becomes better than the *x* channel, as seen in Figure A3, when the transmitter is close to the *y* axis. This is because in this case, SNRs of the *x* and *y* channels are changed to 2 and 12.7 dB, respectively, due to the change in the transmitter position. Additionally, we observe that in both figures, the multichannel combining method performs better than each individual channel.
